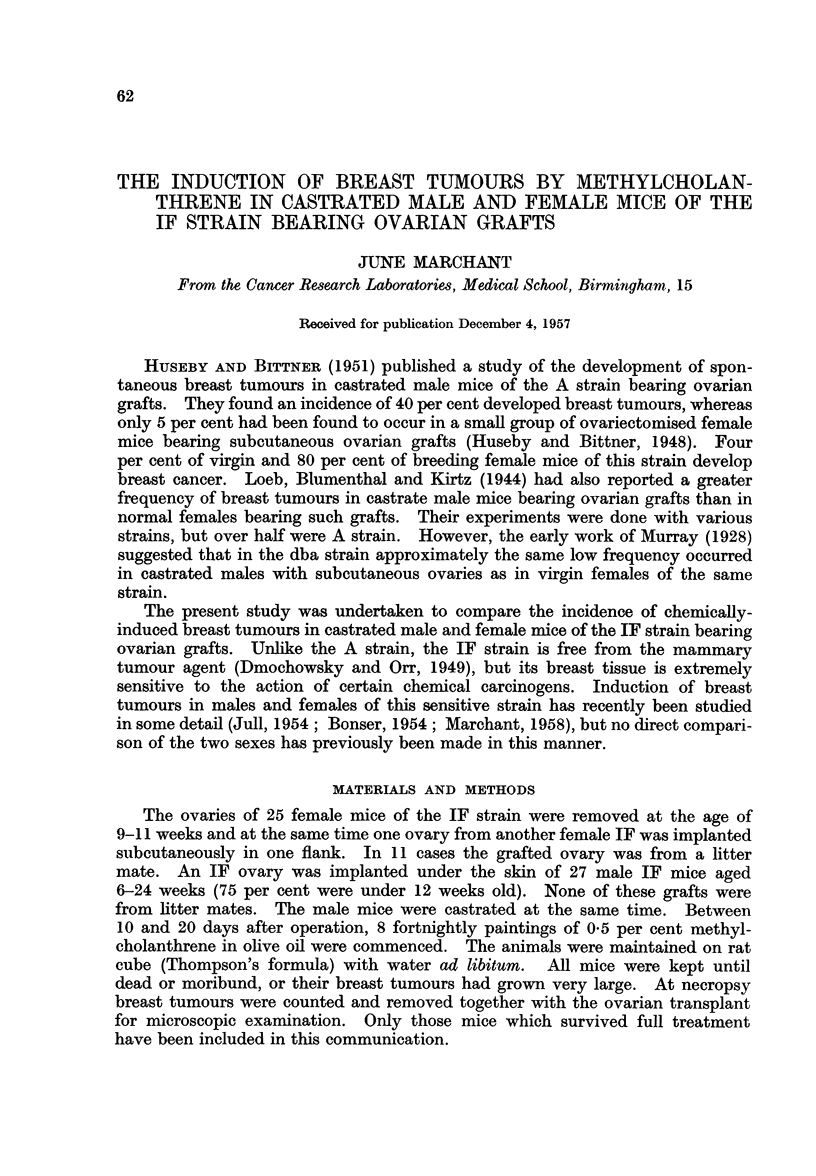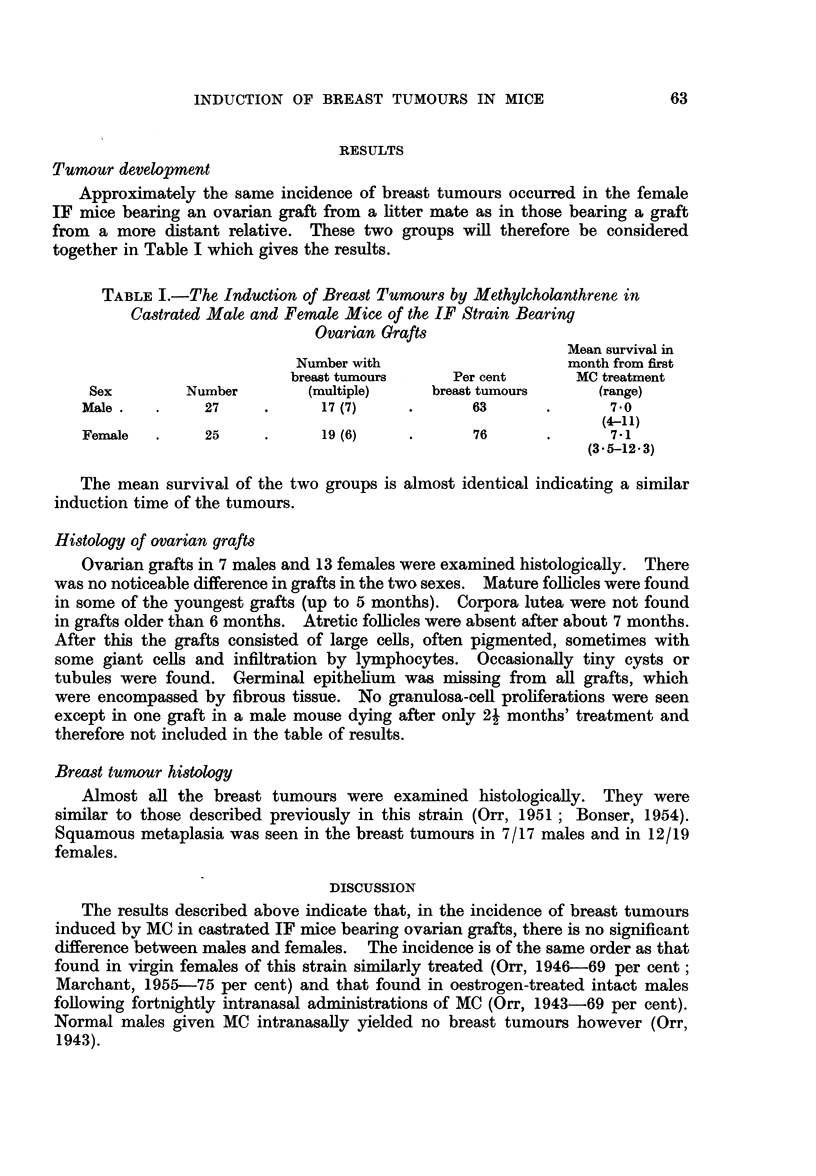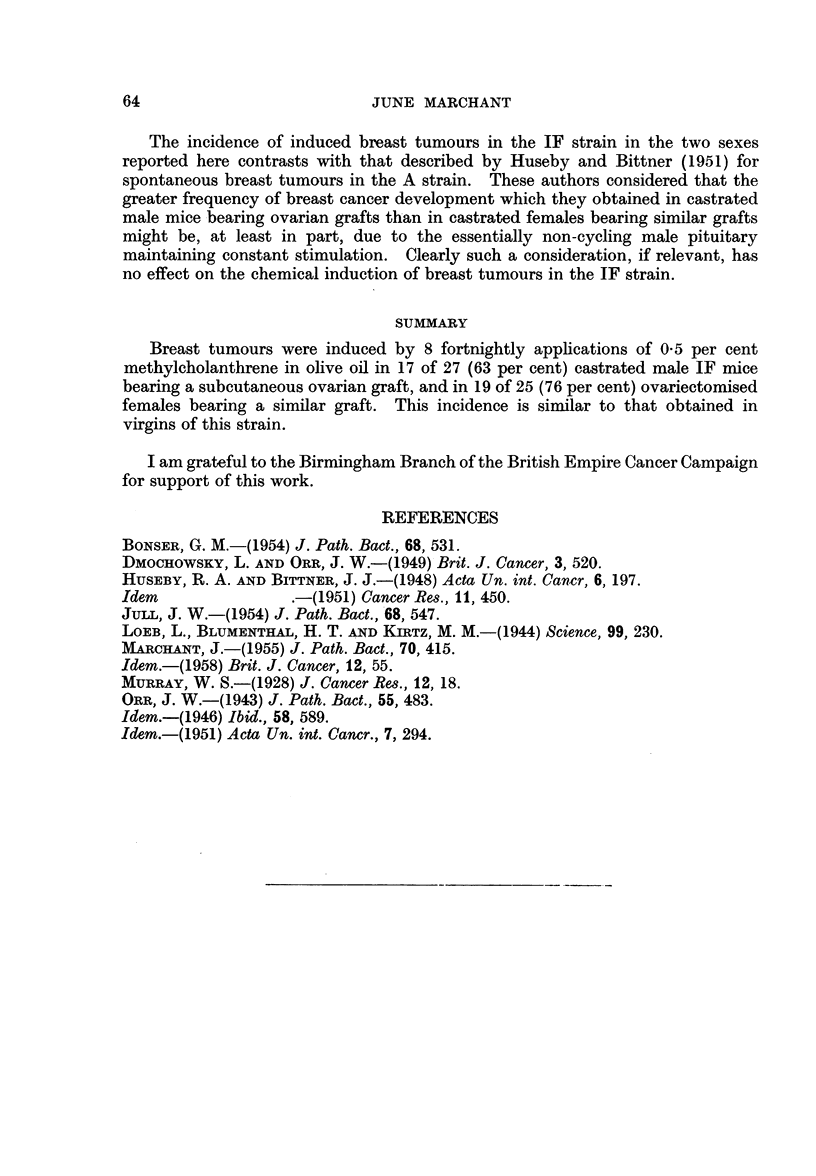# The Induction of Breast Tumours by Methylcholanthrene in Castrated Male and Female Mice of the IF Strain Bearing Ovarian Grafts

**DOI:** 10.1038/bjc.1958.9

**Published:** 1958-03

**Authors:** June Marchant


### THE INDUCTION OF BREAST TUMOURS BY METHYLCHOLANTHRENE IN CASTRATED MALE AND FEMALE MICE OF THE IF STRAIN BEARING OVARIAN GRAFTS

JUNE MARCHANT

*From the Cancer Research Laboratories, Medical School, Birmingham,* 15

Received for publication December 4, 1957

HUSEBY AND BITTNER (1951) published a study of the development of spontaneous breast tumours in castrated male mice of the A strain bearing ovarian grafts. They found an incidence of 40 per cent developed breast tumours, whereas only 5 per cent had been found to occur in a small group of ovariectomised female mice bearing subcutaneous ovarian grafts (Huseby and Bittner, 1948). Four per cent of virgin and 80 per cent of breeding female mice of this strain develop breast cancer. Loeb, Blumenthal and Kirtz (1944) had also reported a greater frequency of breast tumours in castrate male mice bearing ovarian grafts than in normal females bearing such grafts. Their experiments were done with various strains, but over half were A strain. However, the early work of Murray (1928) suggested that in the dba strain approximately the same low frequency occurred in castrated males with subcutaneous ovaries as in virgin females of the same strain.

The present study was undertaken to compare the incidence of chemically-induced breast tumours in castrated male and female mice of the IF strain bearing ovarian grafts. Unlike the A strain, the IF strain is free from the mammary tumour agent (Dmochowsky and Orr, 1949), but its breast tissue is extremely sensitive to the action of certain chemical carcinogens. Induction of breast tumours in males and females of this sensitive strain has recently been studied in some detail (Jull, 1954 ; Bonser, 1954 ; Marchant, 1958), but no direct comparison of the two sexes has previously been made in this manner.

### MATERIALS AND METHODS

The ovaries of 25 female mice of the IF strain were removed at the age of 9–11 weeks and at the same time one ovary from another female IF was implanted subcutaneously in one flank. In 11 cases the grafted ovary was from a litter mate. An IF ovary was implanted under the skin of 27 male IF mice aged 6–24 weeks (75 per cent were under 12 weeks old). None of these grafts were from litter mates. The male mice were castrated at the same time. Between 10 and 20 days after operation, 8 fortnightly paintings of 0·5 per cent methylcholanthrene in olive oil were commenced. The animals were maintained on rat cube (Thompson's formula) with water *ad libitum.* All mice were kept until dead or moribund, or their breast tumours had grown very large. At necropsy breast tumours were counted and removed together with the ovarian transplant for microscopic examination. Only those mice which survived full treatment have been included in this communication.

### RESULTS

### *Tumour development*

Approximately the same incidence of breast tumours occurred in the female IF mice bearing an ovarian graft from a litter mate as in those bearing a graft from a more distant relative. These two groups will therefore be considered together in Table I which gives the results.

TABLE I.—*The Induction of Breast Tumours by Methylcholanthrene in Castrated Male and Female Mice of the IF Strain Bearing Ovarian Grafts*

| Sex | Number | Number with breast tumours (multiple) | Per cent breast tumours | Mean survival in month from first MC treatment (range) |
|---|---|---|---|---|
| Male | 27 | 17 (7) | 63 | 7·0 (4–11) |
| Female | 25 | 19 (6) | 76 | 7·1 (3·5–12·3) |

The mean survival of the two groups is almost identical indicating a similar induction time of the tumours.

### *Histology of ovarian grafts*

Ovarian grafts in 7 males and 13 females were examined histologically. There was no noticeable difference in grafts in the two sexes. Mature follicles were found in some of the youngest grafts (up to 5 months). Corpora lutea were not found in grafts older than 6 months. Atretic follicles were absent after about 7 months. After this the grafts consisted of large cells, often pigmented, sometimes with some giant cells and infiltration by lymphocytes. Occasionally tiny cysts or tubules were found. Germinal epithelium was missing from all grafts, which were encompassed by fibrous tissue. No granulosa-cell proliferations were seen except in one graft in a male mouse dying after only 2½ months' treatment and therefore not included in the table of results.

### *Breast tumour histology*

Almost all the breast tumours were examined histologically. They were similar to those described previously in this strain (Orr, 1951 ; Bonser, 1954). Squamous metaplasia was seen in the breast tumours in 7/17 males and in 12/19 females.

### DISCUSSION

The results described above indicate that, in the incidence of breast tumours induced by MC in castrated IF mice bearing ovarian grafts, there is no significant difference between males and females. The incidence is of the same order as that found in virgin females of this strain similarly treated (Orr, 1946—69 per cent ; Marchant, 1955—75 per cent) and that found in oestrogen-treated intact males following fortnightly intranasal administrations of MC (Orr, 1943—69 per cent). Normal males given MC intranasally yielded no breast tumours however (Orr, 1943).

The incidence of induced breast tumours in the IF strain in the two sexes reported here contrasts with that described by Huseby and Bittner (1951) for spontaneous breast tumours in the A strain. These authors considered that the greater frequency of breast cancer development which they obtained in castrated male mice bearing ovarian grafts than in castrated females bearing similar grafts might be, at least in part, due to the essentially non-cycling male pituitary maintaining constant stimulation. Clearly such a consideration, if relevant, has no effect on the chemical induction of breast tumours in the IF strain.

### SUMMARY

Breast tumours were induced by 8 fortnightly applications of 0·5 per cent methylcholanthrene in olive oil in 17 of 27 (63 per cent) castrated male IF mice bearing a subcutaneous ovarian graft, and in 19 of 25 (76 per cent) ovariectomised females bearing a similar graft. This incidence is similar to that obtained in virgins of this strain.

I am grateful to the Birmingham Branch of the British Empire Cancer Campaign for support of this work.

### REFERENCES

Bonser, G. M.—(1954) *J. Path. Bact.*, **68**, 531.
Dmochowsky, L. and Orr, J. W.—(1949) *Brit. J. Cancer*, **3**, 520.
Huseby, R. A. and Bittner, J. J.—(1948) *Acta Un. int. Cancr*, **6**, 197.
*Idem* .—(1951) *Cancer Res.*, **11**, 450.
Jull, J. W.—(1954) *J. Path. Bact.*, **68**, 547.
Loeb, L., Blumenthal, H. T. and Kirtz, M. M.—(1944) *Science*, **99**, 230.
Marchant, J.—(1955) *J. Path. Bact.*, **70**, 415.
*Idem*.—(1958) *Brit. J. Cancer*, **12**, 55.
Murray, W. S.—(1928) *J. Cancer Res.*, **12**, 18.
Orr, J. W.—(1943) *J. Path. Bact.*, **55**, 483.
*Idem*.—(1946) *Ibid.*, **58**, 589.
*Idem*.—(1951) *Acta Un. int. Cancr.*, **7**, 294.